# A Narrative Review of Digital Addiction and Health: A New Challenge for Modern Medicine

**DOI:** 10.7759/cureus.100491

**Published:** 2025-12-31

**Authors:** Dipesh Govindbhai Patel, Abhishek Hanumanpratap Singh Kshatri, Sahitya Kommuru, Chaitanya Kumar Javvaji

**Affiliations:** 1 Psychiatry, General Hospital, Mehsana, IND; 2 Emergency Medicine, Apollo Hospitals Health City, Visakhapatnam, IND; 3 Public Health, Tufts School of Medicine, Boston, USA; 4 Pediatrics, Jawaharlal Nehru Medical College, Datta Meghe Institute of Higher Education and Research, Wardha, IND

**Keywords:** digital detox, gaming disorder (gd), neurobiology of addiction, public mental health, social media addiction

## Abstract

Digital addiction, encompassing problematic use of the internet, smartphones, gaming, and social media, has emerged as a growing global public health concern. This narrative review synthesises current evidence on the epidemiology, neurobiological underpinnings, diagnostic challenges, health consequences, and management strategies for digital addiction. Prevalence estimates vary widely, with higher rates reported in low- and middle-income countries and among adolescents and university students. Neuroimaging and neurochemical studies demonstrate alterations in reward pathways and prefrontal control networks, mirroring mechanisms seen in substance use disorders (SUDs). Clinically, digital addiction contributes to sleep disruption, visual strain, musculoskeletal problems, psychiatric comorbidities, impaired academic and occupational functioning, and increased risk behaviours. Diagnosis remains challenging due to the absence of universally accepted criteria, though instruments such as Young’s Internet Addiction Test (IAT), the Smartphone Addiction Scale (SAS), and newer culturally adapted tools show promise. Management approaches include early educational interventions, parental strategies, cognitive-behavioural therapy, mindfulness-based interventions, pharmacological options in select cases, and digital detox programmes, though evidence for long-term efficacy remains limited. Emerging artificial intelligence-driven psychoinformatics offers novel opportunities for early detection and personalised intervention. Overall, digital addiction represents a behavioural addiction with substantial biopsychosocial impacts, warranting greater clinical recognition, policy attention, and high-quality research to establish effective, scalable prevention and treatment strategies.

## Introduction and background

Digital addiction is broadly defined as the persistent, compulsive use of digital technologies, including the internet, smartphones, video games, and social media, to the extent that it significantly interferes with daily life. It exhibits core behavioural features common to other addictions, such as impaired control over use, growing tolerance, the experience of withdrawal symptoms when use is restricted, and continued use despite negative consequences [1]. This umbrella term encompasses specific subtypes like internet addiction, smartphone addiction, gaming addiction, and social media addiction. For example, social media addiction often manifests as a compulsive urge to check or update online profiles repeatedly, even when such behaviour undermines personal or professional responsibilities [2]. 

While moderate engagement with digital media can be benign or beneficial, health authorities have outlined guidelines to help identify excessive use. For instance, the American Academy of Paediatrics (AAP) previously recommended a maximum of two hours per day of recreational screen time for children and adolescents; however, recent guidance has shifted away from strict time limits in favour of a more individualised, quality-focused approach [3]. In 2023, the AAP introduced the “5 C’s” framework: considering the Child’s needs, the Content being consumed, opportunities for Calm without devices, what screen time is Crowding Out, and open Communication about use, to encourage healthy digital habits instead of rigid screen-time caps [3]. Consistently exceeding reasonable use (especially if accompanied by signs like loss of control over device use or distress when not online) may signal digital addiction. The World Health Organisation (WHO) likewise advises limiting sedentary screen exposure in youth as part of its global recommendations to reduce sedentary behaviour [4]. In adults, habitual non-work screen time beyond roughly two to three hours per day has been linked to adverse outcomes, including poorer sleep, higher stress levels, and increased depressive symptoms [5].

The concept of digital addiction has evolved significantly since the mid-1990s. Psychiatrist Ivan Goldberg is credited with coining the term “Internet Addiction Disorder” in 1995, initially as a satirical commentary, yet it quickly attracted genuine academic interest [6]. Soon after, Kimberly Young published pioneering work defining internet addiction as a clinical disorder, drawing parallels to pathological gambling [7]. Around the same time, Mark Griffiths and others expanded the discussion by characterising excessive video gaming and computer use as forms of technological addiction, laying conceptual foundations that remain influential in contemporary research [8].

In recent years, the clinical recognition of digital addictions has progressed in some areas, albeit unevenly. The WHO included “Gaming Disorder” in the International Classification of Diseases, Eleventh Revision (ICD-11) as an official diagnosis, characterised by impaired control over gaming, prioritisation of gaming over other interests, and continuation despite negative outcomes, with functional impairment evident for at least 12 months [9]. By contrast, the American Psychiatric Association’s Diagnostic and Statistical Manual of Mental Disorders, Fifth Edition (DSM-5), does not formally recognise most digital-related addictions; it includes internet gaming disorder only as a condition for further study rather than as an official diagnosis [10]. This discrepancy reflects how only certain subtypes, gaming disorder in particular, have attained formal diagnostic status, while others (such as problematic smartphone use or social media overuse) remain under debate and lack consensus criteria [11].

Prevalence rates of digital addiction vary widely across different regions and demographic groups. In East Asian countries, notably China and South Korea, adolescent internet addiction is often reported in about 10% or more of youths [12]. South Korea, for example, has long viewed digital addiction as a serious public health issue, establishing specialised treatment centres and intervention programs. In India, recent systematic reviews and meta-analyses have found that approximately 19.9% of college students meet criteria for internet addiction using strict cut-offs on Young’s Internet Addiction Test (IAT) and that this prevalence can rise to about 40.7% under more inclusive criteria [13]. By contrast, studies in Western countries such as the United States and Germany typically report lower prevalence, often below 8% of the population, although even in these settings, a significant minority engage in problematic digital use [14].

The COVID-19 pandemic further amplified concerns around digital overuse. During lockdowns and social distancing measures, screen time increased globally as people increasingly turned to digital devices for work, schooling, communication, and entertainment. Many individuals developed more severe or frequent patterns of problematic digital use during this period, exacerbating pre-existing vulnerabilities [15]. Consequently, numerous reports documented increases in internet and gaming addiction globally in 2020-2021, accompanied by parallel rises in related psychological distress such as heightened anxiety, depression, and sleep disturbances [16].

Given these developments, this narrative review aims to provide a comprehensive overview of current knowledge on digital addiction, including its epidemiology, clinical characteristics, underlying neurobiology, diagnostic challenges, and strategies for prevention and management. Notably, the evidence base for treating digital addiction remains nascent. A recent umbrella review of meta-analyses concluded that most interventions, from cognitive-behavioural therapy and counselling to exercise programs, have weak or inconsistent efficacy, underscoring the need for more robust research [17]. We emphasise the implications for public health policy, medical practice, and education, intending to guide clinicians, policymakers, and educators in effectively recognising and managing digital addiction in today’s increasingly digitised society. Digital addiction is classified as an impulse control disorder that involves the obsessive use of digital technologies and platforms. The main purpose of this narrative review is to identify the risk factors and vulnerable population, understand existing treatment options, explore the role of artificial intelligence tools, and highlight gaps and opportunities for future research. 

## Review

Epidemiology and risk factors

Digital addiction can manifest in several forms, including social media addiction, gaming addiction, smartphone addiction, online compulsions, compulsive information seeking, and porn and cybersex addiction [18]. Globally, 5.56 billion people use the internet, accounting for 67.9% of the world’s total population. A study published in the Journal of Addiction by Acharya et al. in 2023 states that globally, there were 5.32 billion mobile users and 4.65 billion social media users in 2022, and this number continues to rise, with a 4.1% increase in social media users each year [19]. Although internet addiction is one of the most prevalent forms of addiction, it is important to understand that digital addiction doesn’t always need internet usage, and it accounts for offline activities such as gaming, online relationships, and compulsive browsing [20]. It is essential to broaden our attention to other forms as well. Existing literature states that about 1/4^th^ of the general population in the world could be affected by one or the other form of digital addiction [20].

In their meta-analysis of 507 studies and about 200,000 individuals from 64 countries, Meng et al. reported pooled global prevalence rates of 26.99% (95% CI 22.73-31.73) for smartphone addiction, 17.42% (95% CI 12.42-23.89) for social media addiction, 14.22% (95% CI 12.90-15.65) for internet addiction, 8.23% (95% CI 5.75-11.66) for cybersex addiction, and 6.04% (95% CI 4.80-7.57) for game addiction, all statistically significant [20]. A more recent meta-analysis focusing on university students across 38 countries (Liu et al., 2025) found an even higher pooled prevalence of internet addiction at 41.84% (95% CI 35.89-48.02), with male students at significantly higher risk than female students (pooled OR 1.32, 95% CI 1.19-1.46) [21].

Meng et al. also found that the prevalence of digital addiction was higher in low- and lower-middle-income countries [20]. Another systematic review across 31 countries stated that the prevalence of internet addiction was threefold higher than that of pathological gambling, another impulse control disorder, with North America having the highest regional prevalence of 8% [22]. In a publication by the U.S. Naval Institute, it was reported that 78% of adolescents in the US between the ages of 18 and 24 were relying on social media, online dating, video games, and online pornography to such an extent that digital technology could be considered “the new drug” [23]. This trend has been ongoing for the last two decades and particularly worsened during the COVID-19 pandemic [20,24]. 

Previous studies have also shown that gender-related differences exist in most addictive behaviours [20]. These differences are attributed to social factors, biological responses, and co-occurring psychiatric disorders [25]. Several meta-analyses have found that males are more prone than females to engage in addictive behaviours, including digital addiction [25-27]. This trend is likely because males tend to use applications with higher addiction potential, such as gaming and cybersex [25], and because they have fewer protective mechanisms like effortful control and higher tendencies to engage in risky behaviours such as maladaptive cognitions [25,28]. While this continues to be the dominant pattern, some studies report exceptions; for example, research in China, Turkey, and the USA has shown slightly higher rates among female schoolchildren [29,30]. Existing literature also indicates that personality traits, familial factors, alcohol use, and co-existing affective disorders such as depression and anxiety are among the strongest risk factors for digital addiction [31].

Pathophysiology of digital addiction

Digital addiction is an umbrella term encompassing excessive internet use, online gaming, smartphone overuse, and social media addiction, all characterised by compulsive engagement despite negative consequences [32]. Individuals with digital addiction often develop tolerance (needing more digital stimulation for the same effect) and experience withdrawal symptoms (e.g., irritability or anxiety) when cut off, analogous to substance withdrawal [33,34]. They typically struggle to control their usage and continue despite impairments in daily functioning [33,34]. Adolescents and young adults are especially vulnerable due to developmental neurobiological factors and widespread access to digital devices [33,34]. Neurobiologically, digital addictive behaviours engage brain reward and stress pathways in ways similar to drugs of abuse, inducing neural adaptations over time [35]. Chronic overuse of digital media can alter neurotransmitter systems (notably dopamine) and neural circuitry involved in mood and impulse control [36,37]. Consistent with this, animal models of “gaming disorder” have demonstrated addiction-like behaviour (e.g., loss of control over reward-seeking) and altered neural activity in reward-related regions (prefrontal cortex (PFC), nucleus accumbens, amygdala, etc.) after excessive digital gameplay [38]. These findings highlight that digital addiction is a bona fide behavioural addiction with a pathophysiology mirroring other addictions in many key aspects [35]. Recent research reveals unique mechanisms specific to digital behavioural addictions that differ from classical substance use disorders (SUDs) [32,39]. Adolescents are particularly vulnerable due to their ongoing brain development, heightened neuroplasticity, and increased sensitivity of the reward system to environmental stimuli. The immature PFC, combined with a hyper-responsive limbic system, creates a developmental window of susceptibility to addictive behaviours. Recent studies also show neurotransmitter dysregulation beyond dopamine, including elevated GABA levels in the anterior cingulate cortex and disrupted glutamate pathways [40].

Brain Reward Systems in Digital Addiction

**Figure 1:**
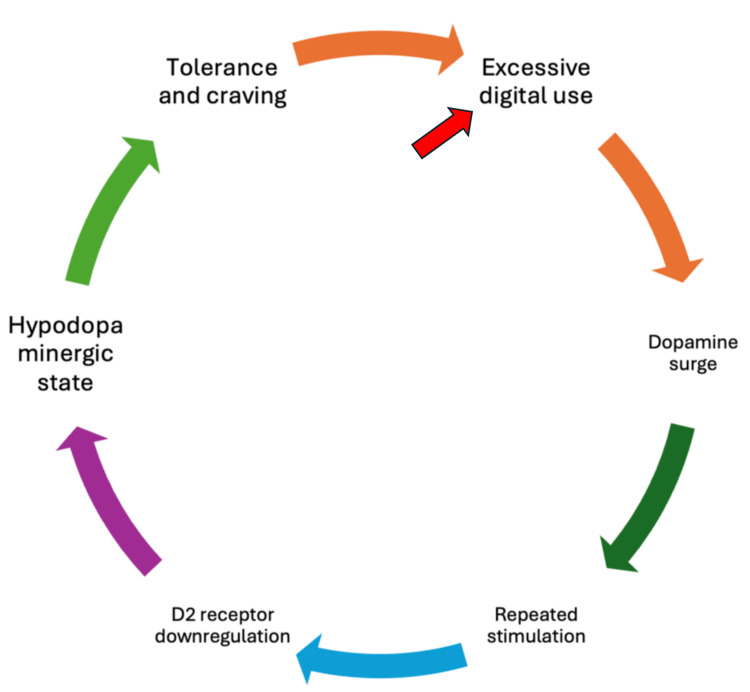
Brain reward cycle in digital addiction D2: dopamine receptor D2; the red arrow indicates the starting point. Created by author, Chaitanya Kumar Javvaji.

Digital addictions hijack the brain’s natural reward circuitry, centred on the mesolimbic dopamine system, in a manner analogous to substance addictions [35]. Functional neuroimaging and neurotransmitter studies indicate that engaging in online gaming or other digital rewards triggers dopamine release in the striatum comparable to psychoactive drugs. For example, video game play can elevate striatal dopamine to levels seen with stimulant drugs, and repeated binge-playing is associated with downregulation of dopamine D₂ receptors and transporters, reflecting a blunted dopaminergic response similar to chronic drug use [36,41]. Figure 1 shows the brain reward cycle in digital addiction.

Individuals with IGD have shown reduced dopamine receptor availability in the dorsal striatum on PET scans, consistent with dopamine desensitisation in the reward pathway [37]. Cue-reactivity studies further support overlap with drug addiction: exposure to game-related or social media cues activates classic reward regions such as the ventral striatum, amygdala, and ventromedial PFC, correlating with subjective craving [35,42]. In excessive social media users, recent PET evidence found that greater daily social app use correlates with lower dopamine synthesis capacity in the bilateral putamen, suggesting that heavy digital social stimulation may induce dopaminergic downregulation as the brain adapts [43]. Beyond dopamine, other limbic regions involved in reward and emotion show alterations. The amygdala, for instance, exhibits structural and functional changes in digital addicts (e.g., smaller grey matter volume in social networking addicts), reflecting a hyper-reactive yet “efficient” impulsive reward system [44]. Overall, digital addictions tap into the same dopamine-driven reward circuit that underlies drug reinforcement, explaining the strong reinforcing properties of online games, apps, and social media and the compulsive “wanting” they provoke. Importantly, this reward hyperactivation is coupled with neuroadaptations (receptor downregulation, etc.) that can leave digital addicts in a hypodopaminergic state, driving further compulsive use in pursuit of normal pleasure levels [36,41].

Neuroimaging Findings in Digital Addiction

**Figure 2:**
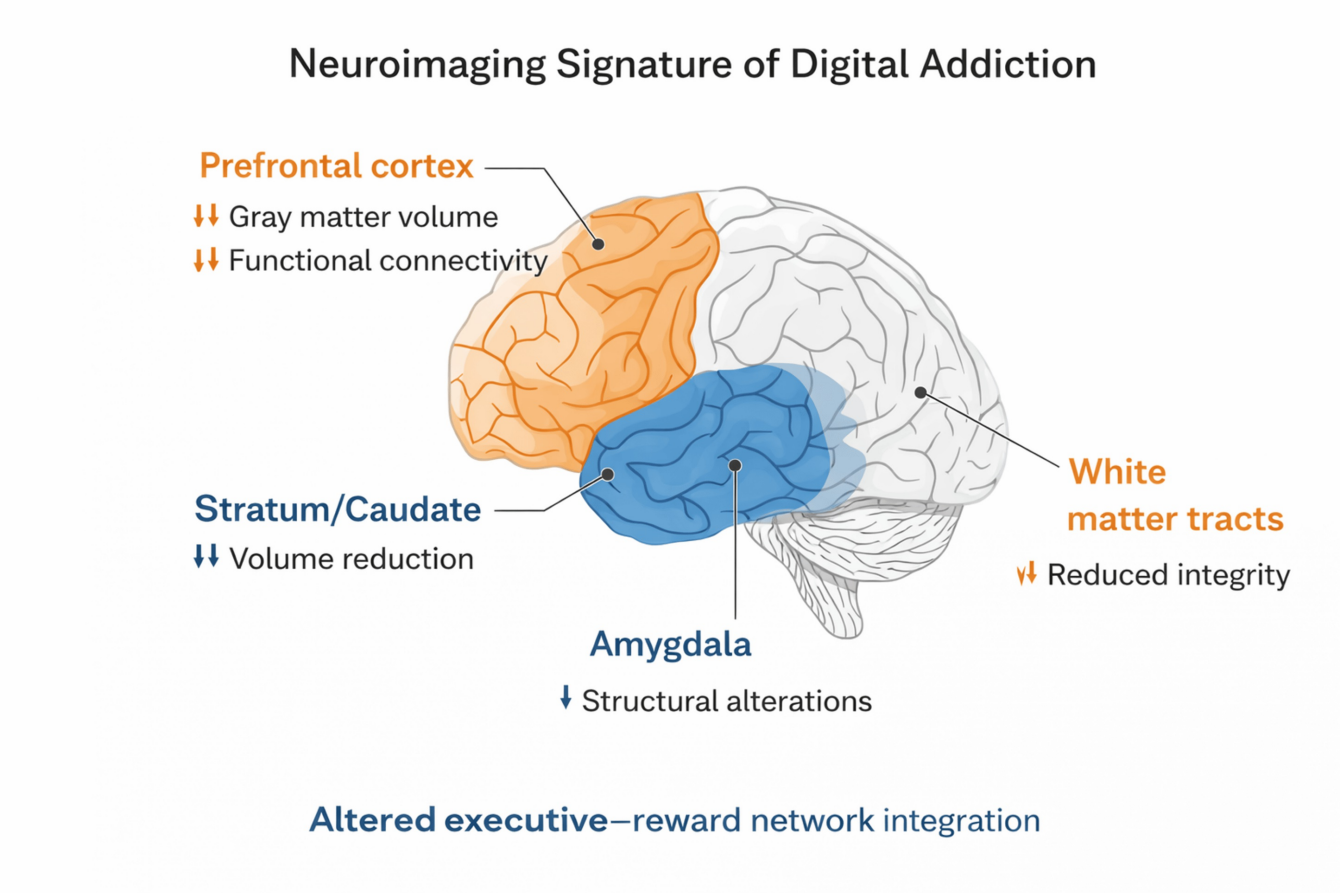
Neuroimaging signature of digital addiction Created by author, Chaitanya Kumar Javvaji.

Neuroimaging studies consistently show that digital addiction is associated with both functional and structural brain changes, especially in prefrontal and reward-related regions [32,35]. The PFC, critical for impulse control and decision-making, is one of the most affected areas. Users with internet or gaming addiction display reduced activity and connectivity in the dorsolateral and orbitofrontal PFC during cognitive tasks, alongside deficits in inhibitory control [35,45]. Grey matter volume reductions in prefrontal regions have been documented in adolescents and young adults with problematic internet/gaming use [45,46]. For example, voxel-based morphometry analyses found lower volume in the inferior frontal gyrus, premotor cortex, and anterior cingulate cortex proportional to internet addiction severity [47]. Consistent with these findings, a recent scoping review identified the prefrontal lobes as the region most consistently impacted across all forms of digital addiction [32]. Besides frontal lobe changes, digital addiction entails abnormalities in limbic and subcortical structures. Neuroimaging of individuals with IGD or smartphone addiction has shown reduced volumes in the striatum (including ventral striatum and caudate) and insula, regions involved in reward processing, habit formation, and interoceptive awareness [32,46]. Notably, adolescents with heavy smartphone use exhibited smaller caudate nucleus volumes than controls, paralleling findings in drug addictions where striatal atrophy accompanies prolonged dopamine overstimulation [32,48]. Similarly, social media addicts had significantly reduced bilateral amygdala volumes, indicating alterations in emotion/reward circuits [44]. Functional connectivity (FC) studies reveal that digital addiction disrupts large-scale brain networks. Resting-state functional MRI (fMRI) analyses have found reduced connectivity within the executive control network and heightened connectivity in salience/reward networks in those with internet or gaming addiction. Such FC impairments are especially noted between prefrontal control regions and subcortical areas, suggesting weakened top-down regulation over impulsive drives [35]. Indeed, neuropsychological testing corroborates poorer decision-making and executive function in IGD, correlating with PFC hypoactivation on fMRI [35,45]. There is also evidence of reduced white-matter integrity in prefrontal and limbic pathways in internet addicts, implying structural connectivity deficits in self-control circuits [36]. In summary, neuroimaging converges on a profile of digital addiction involving a compromised prefrontal “brake” system (smaller, less active PFC and anterior cingulate) alongside an over-responsive reward system (striatal and limbic changes), as well as alterations in sensory and temporal regions that may reflect excessive multitasking and salience of online stimuli [45,47]. These neural alterations mirror those seen in other addictions and underpin the clinical symptoms of impaired control, salience of digital cues, and mood dysregulation in affected individuals [45,46]. Machine learning models trained on resting-state EEG data have successfully classified individuals with internet addiction based on altered FC, underscoring the distinct neural signature of digital behavioural addictions [49]. Figure 2 shows the neuroimaging signature of digital addiction.

Comparison with SUDs

Digital addictions show striking neurobiological parallels with SUDs, though some differences exist. Like drugs of abuse, addictive digital behaviours (gaming, social media, etc.) intensely activate the brain’s mesolimbic reward pathway, causing surges in dopamine and associated euphoria [41]. Over time, both lead to adaptive downregulation of the dopamine system; for instance, chronic gamers and internet addicts exhibit reduced striatal D₂ receptor levels and blunted dopaminergic signalling similar to cocaine or methamphetamine abusers [37,41]. In both SUD and digital addiction, cues related to the addictive stimulus trigger heightened activity in reward-related regions (e.g., nucleus accumbens, amygdala) and craving responses, as shown by neuroimaging studies of gaming or social media cues that mirror drug-cue reactivity [32,42]. Furthermore, individuals with digital addiction and those with substance dependence share patterns of PFC deficits, including reduced grey matter volume and hypofunction in the dorsolateral PFC, ventromedial PFC, and anterior cingulate, underlying poor impulse control and decision-making [45,46]. A systematic review concluded that internet gaming addicts have “deficient neuronal reward systems” and impaired executive control networks akin to those found in drug addiction, supporting the view that both conditions may share a common addiction syndrome [37,45]. Notably, brain imaging has even been used to successfully distinguish IGD patients from healthy individuals using machine-learning models, underscoring that IGD produces robust and unique neural signatures much like substance addiction [41]. Despite these similarities, there are also distinctions between digital and substance addictions. Importantly, digital addiction lacks direct pharmacological toxicity: there is no exogenous drug flooding the brain, so physical health consequences tend to be indirect (e.g., sleep deprivation, sedentary effects) rather than organ [34,44]. Neuroimaging hints at nuanced differences in neural involvement, for example, one study found that social network addicts did not show the reduced anterior cingulate volume typically seen in drug addicts, suggesting relatively preserved prefrontal monitoring in some forms of digital addiction [44]. Likewise, a direct comparison of smokers vs. internet gaming addicts showed both groups shared diminished prefrontal-striatal connectivity, but with some network differences specific to each addiction type [41]. Clinically, withdrawal symptoms in digital addiction are mostly affective (irritability, cravings) and lack the severe physical withdrawal of substances, though they can still be debilitating [33]. In sum, digital addictions and SUDs exhibit convergent neurobiology, involving dopaminergic dysregulation, fronto-limbic circuit impairment, and neuroplastic changes from chronic stimulation, which explains why behaviours like gaming can become compulsive and hard to quit just as drugs are [41,45]. However, subtle neuroanatomical differences and the absence of a foreign substance’s direct effects mark digital addiction as a “behavioural” addiction, albeit one that engages the same brain disease mechanisms of addiction. This overlap suggests that strategies effective in SUD (e.g., enhancing prefrontal control and reducing cue reactivity) may also benefit digital addiction [36,45]. even as treatment must be tailored to the unique psychosocial triggers of the digital context. Modern digital environments, characterised by algorithm-driven personalisation, infinite scrolling, and intermittent reinforcement through likes and notifications, intensify addictive engagement. These features mimic operant conditioning paradigms, continuously stimulating the brain’s reward circuits [50]. Table 1 shows a comparison of digital addiction vs SUD.

**Table 1:** Comparison between digital addiction and substance use disorder

Category	Digital Addiction	Shared Mechanisms	Substance Use Disorder
Pharmacological toxicity	No pharmacological toxicity	—	Pharmacological toxicity present
Physical harm	Indirect physical harm (sleep disturbance, sedentary lifestyle)	—	Direct organ damage
Dopaminergic function	—	Dopaminergic dysregulation	—
Fronto-limbic circuitry	—	Fronto-limbic impairment	—
Cue processing	—	Cue reactivity	—
Neuroplasticity	—	Neuroplastic changes	—
Overall network effect	—	Altered executive–reward circuitry	—

Physical and mental health consequences 

Digital addiction, defined as excessive use of digital devices and online platforms, including social media, gaming, and over-the-top (OTT) streaming platforms [20,51], plainly interrupts the normal sleep-wake cycle. The blue light and continuous disruption from these digital devices frequently result in the establishment of insomnia and other sleep-onset and sleep-maintenance problems. The underlying physiological mechanism is a chronic hyperarousal state created by the continuous use of digital devices during both day and night [1]. This higher degree of physiological and cognitive stimulation directly counteracts the parasympathetic dominance and consequent relaxation required for restorative sleep initiation and maintenance [52]. Prolonged smartphone and other digital device use significantly exacerbates the constant exposure of light to the eyes that can cause decreased blink rates and increased ocular surface exposure to light and dirt particles, which leads to unstable tear films and evaporative dry eye that give rise to digital eye strain and dry eye disease, which have been rendered worse by the COVID-19 pandemic. These conditions not only cause eye problems, but they are also strongly associated with detrimental effects on mental health, such as stress, anxiety, and depression, creating a vicious cycle [53,54].

Abnormal spine curvatures, notably an exacerbation in the angles of thoracic kyphosis and lumbar lordosis, as well as forward head position [55], are strongly associated with increased smartphone addiction in teenage men with an ectomorphic somatotype. This positive correlation points to a dose-response connection in which higher levels of smartphone addiction are directly linked with a steady rise in these posture abnormalities. These results highlight the fact that improper and extended mobile phone use leads to the development of abnormalities in the spinal column [56]. Overuse of phones can interfere with real-world social relationships, increasing feelings of loneliness and decreasing social support. This has been identified as mobile phone addiction (MPA). Those who suffer from depression and anxiety, on the other hand, might use their phones as a coping method. This leads to a reciprocal relationship that creates a vicious cycle with MPA. The association between excessive phone use and increased anxiety and depressed symptoms is further mediated by poor sleep quality, which is impacted by MPA [57].

Internet and social media platforms, as visual communication tools, encourage users to share photos. However, when young women are exposed to glorified body ideals in the media, they might develop unrealistic body views and create unreasonable expectations. Therefore, the ideal body standards displayed in the media can pose a risk of developing a negative body image for individuals who cannot attain them [58]. Frequent television use during childhood and adolescence may be linked to increased concentration problems, poor reading and homework completion, school ignorance, low grades, academic failure, and not pursuing a postsecondary degree [59].

Adolescents with Internet addiction reported higher self-reported attention-deficit hyperactivity disorder (ADHD) symptoms. Teenagers with ADHD, who have trouble controlling themselves, may find the Internet especially appealing due to its instant gratification and interactive features, which could result in addiction. Additionally, adolescents with Internet addiction exhibited higher levels of depression and hostility, with males showing a stronger association with hostility. The cross-sectional design restricts the ability to conclude causality, and the use of self-reported symptoms of ADHD raises the possibility that subsequent studies need to include data from a range of sources [60].

The study conducted by Santini et al. examines the impact of social media dependency, as assessed by the Bergen Social Media Addiction Scale (BSMAS), on mental health and social isolation outcomes using longitudinal data from a sizeable, representative cohort of Danish adults (aged 15-64, enrolled in school or working), gathered in 2020 and 2021. It reveals that signs of social media addiction strongly predict lower mental health (coef=−0.06) and social network size (coef=−0.03) one year later, as well as greater levels of depression (OR=1.06) and isolation (OR=1.14). Those with BSMAS scores indicating addiction (≥19, which impacted 2.3% of the population) were 2.71 times more likely to suffer hopelessness and 4.4 times more likely to experience loneliness, in addition to having fewer social networks and poor mental health. Additionally, the study shows a two-way interaction in which baseline depression and loneliness predict higher levels of social media addiction symptoms, pointing to a vicious cycle in which social isolation exacerbates addictive behaviour, which in turn weakens social bonds and mental health [61].

Although smartphones are useful in emergencies, their widespread use has sparked safety concerns due to their impact on driver concentration and crash risk. Research, including simulator-based studies, has demonstrated that using a cell phone while driving impairs reaction time, increases mental load, and affects vehicle control. However, underreporting and inaccurate data have made it difficult to evaluate phone use during collisions, raising questions about the actual risk and calling for more meticulously designed research conducted in actual settings. An epidemiological study comparing mobile phone users and non-users revealed a relationship between overall cell phone use and increased crash rates, particularly for crashes involving property damage, despite the lack of reliable data on cell phone use while driving [62,63].

The study conducted by Aleankushiu and Radhi examines the relationship between smartphone use and health behaviours related to nutrition, conducted among 368 female secondary school students in Karbala, Iraq. It was discovered that 73.6% of the students had moderate nutrition-related health behaviours (mean score: 38.44±5.470) and 53.5% had moderate digital addiction (mean score: 29.04±5.857). Regression analysis revealed that higher levels of digital addiction predicted worse nutritional practices, and there was a strong negative association between digital addiction and healthy eating behaviours (r = -0.118, p = 0.023). Higher levels of digital addiction and poorer eating habits were associated with factors including owning digital devices and spending more time on them [64]. Meanwhile, 856 teenagers from the Mediterranean region of Turkey participated in the cross-sectional study, which looked into the connections between teenage aggression, digital gaming addiction, and emotional eating.

The Buss-Perry Aggression Questionnaire (BPAQ), the Digital Game Addiction Scale (DGAS-7), the Emotional Eating Scale (EES), and a personal information form were used to gather data. 32.4% of participants in the study conducted by Caner and Evgin reported having a digital gaming addiction, while male adolescents scored higher on the BPAQ, DGAS, and EES. The findings showed a strong correlation between aggressive behaviour, emotional eating, and digital gaming addiction, suggesting that these three variables are important contributors to emotional eating, which raises the risk of obesity in teenagers [65]. Both studies demonstrate how digital addiction has different effects on teenage eating patterns in males and females, leading to consequences including obesity and malnutrition. Therefore, more thorough research is required to fully comprehend these gender-specific effects and their ramifications. Studies conducted over one year revealed that newly licensed nurses who experienced adverse events were significantly more addicted to their phones than those who did not [66]. Although no direct link was found between addictive device use and academic performance, higher self-reported screen time was linked to lower grade averages. These findings suggest that excessive time spent on screens, despite no addiction, could decrease study time and negatively impact academic achievement [67]. Table 2 describes the summary of pathophysiological mechanisms in digital addiction.

**Table 2:** Summary of pathophysiological mechanisms in digital addiction

Mechanism	Description
Compulsive use	Persistent and repetitive digital engagement despite negative consequences, with loss of control over usage.
Tolerance and withdrawal	Increasing digital stimulation is required to achieve the same effect, with withdrawal symptoms such as irritability and anxiety when access is restricted.
Reward system dysregulation	Excessive digital use activates brain reward pathways similar to substance addiction, reinforcing compulsive behaviour.
Dopamine imbalance	Chronic stimulation leads to altered dopamine signalling, receptor downregulation, and reduced reward sensitivity.
Impaired executive control	Reduced prefrontal cortex activity and connectivity result in poor impulse control and decision-making.
Limbic hyper-reactivity	Structural and functional changes in limbic regions (e.g., amygdala, nucleus accumbens) increase craving and cue reactivity.
Developmental susceptibility	Adolescents are more vulnerable due to immature prefrontal regulation and heightened reward sensitivity during brain development.
Neurotransmitter changes	Alterations extend beyond dopamine, including elevated GABA levels and disrupted glutamate pathways.
Network connectivity changes	Reduced connectivity in executive control networks and increased salience/reward network activity weaken top-down regulation.
Reinforcement by digital design	Algorithm-driven features (likes, notifications, infinite scrolling) perpetuate operant conditioning and addictive engagement.

Diagnosis and assessment tools

Research suggests digital addiction, especially internet and smartphone use, lacks universally accepted diagnostic criteria to diagnose in DSM-5 and ICD-11, but IGD is recognised in the DSM-5 for further study. Currently, the evidence leans toward using DSM-5 criteria for IGD, adapted to diagnose and research new digital addiction and related symptoms and mental health disorders, with at least five of nine symptoms needed for diagnosis over 12 months [68].

The Griffiths, Young, and Tao questionnaire to diagnose internet addiction offers valuable frameworks for diagnosing and understanding modern digital addiction, with a shared emphasis on a lack of control over device usage, conflict with one's own emotions and relationships with others, and preoccupation with digital devices. However, their reliance on diagnosing gambling/substance addiction frameworks, lack of precision in diagnosing, and their limited capacity for adaptation highlight gaps in addressing digital addiction. Young’s IAT is the most widely applied nowadays due to its accessibility and adaptability to diagnosis. Griffiths’ model offers theoretical breadth, and Tao’s criteria provide clinical precision [7,69-71].

These days, the SAS is a popular instrument for evaluating smartphone addiction and aiding in the comprehension of more general digital addiction. SAS and its Short Version (SAS-SV) are exceptionally dependable and accurate, especially for adolescents and young people, but there’s a continuing discussion about their diagnostic accuracy [72,73]. The scale has been proven to have strong validity and reliability, which indicates it evaluates problematic smartphone use consistently and accurately. Additionally, the study conducted by Hamamura et al. found that the SAS-SV helps diagnose mental health issues like impulsivity and depression, suggesting that smartphone addiction may have an impact on mental health [74].

The Mobile Phone Use Screening Test (MUST), which was developed to identify mild, moderate, and excessive/problematic mobile phone use, is a valid and dependable instrument for screening excessive mobile phone use in people between the ages of 18 and 40, particularly in India. Its creation is a step toward comprehending and reducing the negative effects of excessive smartphone use on one's mental health and functionality [75]. Using a sample of 4,493 Estonian adolescents, this study, conducted by Seema et al., details the creation and validation of the Digital Addiction Scale for Teenagers (DAST), a 10-item scale intended to evaluate teen digital addiction. The study conducted by Seema et al., which was divided into pre-COVID (N = 1,972) and distance-learning (N = 2,521) samples, comprised a pilot (N = 40) and a main research study. The DAST separates healthy use from addiction by measuring attitudes and behaviours related to digital gadgets. With positive associations with school burnout, learning challenges, and screen usage, and negative correlations with emotional school involvement and life happiness, it showed a consistent one-factor structure. The scale’s discriminant and external validity were supported, identifying four clusters: digitally addicted, excessive users, endangered, and healthy users. The DAST is recommended for psychoeducational assessment and evaluation of health-related digital competence [76]. DAST is a very helpful, culturally appropriate, and trustworthy instrument for screening, research, and intervention in the setting of teen digital addiction. Because it can assist with cross-cultural research, identify at-risk groups, and facilitate customised therapy, it is an essential tool for tackling the psychological and social elements of digital addiction in teens and beyond. By promoting early detection and directing preventive interventions, the DAST supports adolescent welfare in an increasingly digital world [77-79].

This study, conducted by Manara et al., published on May 12, 2024, introduces and validates the Abstinence from Smartphone Scale (ABSS-10), a 10-item psychometric tool designed to measure psychological symptoms (e.g., craving, irritability) associated with smartphone abstinence among university students. In Study 1, university students completed the Acute Behavioral Symptoms of Smartphone Use Scale (ABSS-10) twice during a 2.5-hour restriction period. In Study 2, it was administered three times over a 5-hour restriction period, alongside general state anxiety and smartphone dependence scales. The ABSS-10 showed good psychometric qualities, identifying changes in symptom severity over time and differentiating abstinence symptoms from dependence and anxiety. It is useful in therapeutic settings (finding people for digital detox or therapy) and research (examining smartphone addiction criteria) [80].

A study conducted by Lin et al. developed and validated the Smartphone Addiction Inventory (SPAI), a 26-item self-administered scale adapted from the Chinese Internet Addiction Scale to assess smartphone addiction in Taiwanese young adults (N=283, mean age 22.9±2.0 years, 260 males, 23 females). Exploratory factor analysis identified four factors: compulsive behaviour, functional impairment, withdrawal, and tolerance. The scale showed high internal consistency (Cronbach’s α=0.94 overall; 0.87, 0.88, 0.81, and 0.72 for respective factors) and test-retest reliability (intra-class correlations 0.80-0.91, p<0.001). Correlations with phantom vibration and ringing syndrome supported construct validity. The SPAI is a reliable tool for identifying smartphone addiction, particularly for research and screening in young adult populations [81].

The studies conducted by Lin et al. and Simó-Sanz et al. assessed the SPAI's factor structure and psychometric qualities among Italian university students (N=485, 29.3% male), translated and modified the SPAI into Spanish (SPAI-S), and validated it in a sample of 2,958 Spanish adults at the University of Valencia. The SPAI supports research on problematic smartphone usage and its behavioural effects by being in line with DSM-5 substance abuse criteria, which makes it a useful instrument for evaluating smartphone addiction in European contexts and allowing comparisons with Asian studies [82,83]. SPAI-SF, a shorter version of the SPAI, was created to improve clinical and research effectiveness. The study conducted by Lin YH et al. shortened the original 26-item SPAI while preserving validity and reliability using data from Taiwanese subjects. In order to detect smartphone addiction, a screening cutoff point was developed, which makes the SPAI-SF useful for rapid evaluations in clinical settings and extensive research [84].

A self-report tool to evaluate problematic mobile phone use, or "cell phone addiction", in English-speaking people, the Problematic Use of Mobile Phones (PUMP) Scale was created and validated by the study conducted by Merlo et al. Its single-factor structure showed excellent internal consistency and convergent validity. Merlo et al.'s study small, convenience-based sample (N = 244), potential self-report biases, and the absence of a "gold standard" for diagnosis, necessitating further research with larger, more diverse populations and objective measures [85].

In primary care, the clinical assessment of digital addiction involves the use of screening tools like the IAT and SAS, with a focus on identifying at-risk persons for referral and diagnosing the mental health impact of digital addiction. Standardised surveys like the MUST and PUMP scales, as well as newly developed scales like DAST, ABSS-10, and SPAI, in-depth interviews, Ecological Momentary Assessment (EMA), and technology use are all part of the more comprehensive review process used in psychiatry to ascertain the overall amount of time spent on technology and its effects on behaviour and general health. The overall results suggest that app-based EMA could be an effective and relevant method for further researching and understanding current digital addiction [86].

Management

There are several major physical and mental health implications related to digital addiction. In 2020, the WHO declared that excessive usage of digital technologies has led to problems like inability to manage time, decreased attention span, and disturbed sleep patterns like excessive daytime sleeping and insomnia during the night [1]. Along with these, it has also been found that digital addiction and excessive screen time increase the risk for cardiovascular diseases such as high blood pressure, obesity, low high-density lipoprotein cholesterol, and poor stress regulation due to sympathetic overactivity and dysregulated cortisol production [87]. This section explores current treatment strategies and highlights existing gaps, challenges, and opportunities for future research. 

Early Intervention

Early intervention remains the key aspect of managing digital addiction since milder forms are often more responsive to behavioural strategies and can be addressed before the need for pharmacologic or intensive psychological interventions. Early intervention for digital addiction can be classified into four approaches: 1. Children’s education, 2. Parenting strategy 3. Strategic physical activity, and 4. Counselling [88]. In 2014, a randomised control trial was conducted by Walther, Hanewinkel & Morgenstern in which it was found that four 90-minute classroom sessions covering topics like internet use, online communication, gaming and gambling addiction resulted in significantly lower gaming frequencies and gaming time per day [89]. It was also found that there were significantly lower addiction rates in the intervention group compared to the control group [89]. A study was conducted to assess the role of parental control strategies in managing digital addiction. The Triple P-Positive Parenting Program was another important study focused on improving family dynamics, enhancing parenting skills, and reducing problem behaviours in children. The study conducted by Ozyurt Gin et al. in Turkey was on 76 mother and child pairs, wherein mothers were educated about providing a safe and engaging environment, creating positive learning experiences, consistent discipline, and setting realistic expectations. After this intervention, daily digital device use time decreased significantly from 2.88 ± 1.21 hours to 1.51 ± 0.91 hours (p < 0.001), and frequency of device use per week dropped from 6.92 ± 0.26 days to 5.39 ± 0.94 days (p < 0.001) [90]. 

Self-Control Interventions

Self-control interventions like mindfulness therapy emphasise consciously perceiving and focusing attention on the present, stimulating positive emotions, and maintaining the best state of emotional calm and safety [91-93]. In a randomised control trial conducted by Cheng et al., mindfulness training is beneficial to improve the ability of self-control and reduce rumination levels, thereby inhibiting the negative impact of smartphone addiction [93]. 

Cognitive Behavioural Therapy (CBT)

CBT is the most widely studied and effective intervention for digital addiction. In 2023, Ayub et al. reviewed 10 randomised controlled trials focusing on treating internet addiction in adolescents and young adults. They found that most of these studies showed a reduction of scores on Young’s IAT, weekly time spent online, and even decreased symptoms of depression and anxiety following individual or group-based cognitive behavioural therapy delivered for eight to 12 weeks, with effects lasting up to six weeks [94]. Similar studies were conducted by Young. In 2007, Yamada et al., involving 114 participants and 26 participants, respectively, meeting criteria for internet addiction, showed that individuals completing 12 weeks of CBT for internet addiction demonstrated clinically significant improvement in symptoms, with 78% maintaining the same improvement at the six-month follow-up [95,96]. Studies were also conducted to test the effectiveness of cognitive-behavioural group therapy (CBGT) on internet addictive behaviours, depression, and anxiety.

The study conducted by Ksiksou et al. on 60 nursing students in Morocco randomly assigned them to either the intervention group, who received eight sessions of CBGT, or the control group, and the results were assessed using the IAT and the Depression, Anxiety, and Stress Scale [97]. It showed a statistically significant reduction in internet addiction, depression, and anxiety [97]. Existing literature also has a case report conducted on a 15-year-old adolescent boy with problems of internet, gaming, pornography, information bingeing, and social media addiction. The boy was given 14 sessions of dialectical behavioural therapy covering seven modules, after which there was a significant improvement in his targeted self-regulation behaviours, leading to decreased nighttime screen exposure and decreased use of pornography [98]. Recently, Lu et al. conducted an umbrella review that included five studies assessing eight types of treatments, including group counselling, internet addiction prevention programs, psychosocial interventions, reality therapy, self-control training, cognitive behavioural therapy, programs to reduce screen time in kids, and exercise [17].

They found that CBT reduced anxiety (standardised mean difference (SMD) 0.939, 95% CI 0.311-1.586), IGD symptoms (SMD 1.394, 95% CI 0.664-2.214), gaming time (SMD 1.259, 95% CI 0.311-2.206), and internet addiction scores (SMD -2.097, 95% CI -2.814 to -1.381). Group counselling improved self-control (SMD 1.296, 95% CI 0.269-2.322) and lowered internet addiction (SMD -1.417, 95% CI -1.836 to -0.997). Exercise reduced internet addiction (SMD -2.322, 95% CI -3.212 to -1.431), depression (SMD -1.421, 95% CI -2.046 to -0.797), and interpersonal sensitivity (SMD -1.433, 95% CI -2.239 to -0.627) [17]. Prevention programs and child-focused screen time reduction programs were found to be ineffective, while all the other programs were only moderately effective [17]. 

Pharmacologic Interventions

While early interventions and CBT are widely used methods for managing digital addiction, there is a need for pharmacological interventions with agents like bupropion and selective serotonin reuptake inhibitors in cases with comorbid psychiatric conditions and/or severe behavioural dysregulation. In a six-week trial conducted by Han et al. on gaming addiction patients with comorbid anxiety and high novelty-seeking traits, they found that bupropion significantly reduced gaming time from 36.6 to 18.9 hours and improved internet addiction scores by 27% [99]. In 2016, a study was conducted to compare the effects of bupropion and escitalopram on IGD.

The study was conducted by Song et al. on 119 adolescents for six weeks, and subjects were evaluated using the Clinical Global Impression-Severity (CGI-S) Scale, the Young's IAT, the Beck Depression Inventory (BDI), the ADHD Rating Scale (ARS), and the Behavioral Inhibition and Activation Scale (BIS/BAS) [100]. Both bupropion and escitalopram improved outcomes compared with observation across multiple domains: CGI-S (3.1 vs 3.6 vs 4.5), Young's IAT (42.5 vs 47.8 vs 66.4), BDI (6.5 vs 5.5 vs 9.4), ARS (4.4 vs 7.0 vs 9.4), BIS/BAS (46.0 vs 47.9 vs 51.6), and BIS (13.6 vs 15.8 vs 18.4), with bupropion showing slightly greater benefits in attention and impulse control [100]. It is also important that some reviews in the existing literature say that the effects of bupropion and escitalopram need to be studied in a larger sample size for a longer duration to fully understand the potential of treatment [101].

Digital Detox

"Digital detox" refers to a period when a person refrains from using tech devices such as smartphones, televisions, computers, tablets, and social media sites. This can be achieved by using apps to track usage, setting screen-free time before bed, and turning off notifications [102]. The extent to which digital detox can be used to manage addiction has been studied independently by two groups, arriving at similar conclusions. Both studies stated that there is no clear definition for digital detox and no consistency in the assessment of effectiveness [103,104]. Moreover, the effect of this intervention is highly dependent on the duration of intervention, age, gender, and contextual factors, and is not alone enough in managing addiction [103,104]. Another study was conducted on 467 young adults [105]. Anxiety scores declined from 12.5 to 6.6 in males and from 14.7 to 8.3 in females, while depression decreased across occupational groups, e.g., from 13.5 to 6.8 in full-time employees and 14.0 to 7.1 in unemployed participants [105]. 

Role of Artificial Intelligence and Future Research 

Artificial intelligence is emerging as a promising tool in the detection and management of digital addiction. Montag and Diefenbach introduced the concept of psychoinformatics, where artificial intelligence analyses behavioural data to identify patterns of compulsive technology use and support real-time interventions [106]. Brand et al. expanded on this through the Interaction of Person-Affect-Cognition-Execution (I-PACE) model, emphasising how AI could tailor interventions by integrating individual psychological traits, emotional states, and usage behaviors [107]. Future research should focus on validating these models through longitudinal studies and ensuring ethical, personalised application across diverse populations.

In this narrative review, the primary outcome was to synthesise current evidence on the health implications of digital addiction, including its physical, psychological, neurobiological, and functional consequences. The secondary outcomes included summarising known risk factors and vulnerable populations, describing available diagnostic and screening tools, reviewing current treatment and preventive strategies such as early educational programs, CBT, mindfulness-based interventions, digital detox approaches, and pharmacological options, and exploring the emerging role of artificial intelligence in early detection and personalised intervention. Additionally, this review aimed to identify gaps in existing research, including the lack of standardised diagnostic criteria and limited long-term follow-up data, thereby highlighting areas where future studies are urgently needed.

## Conclusions

Digital addiction is an emerging behavioural disorder with complex biopsychosocial underpinnings and widespread implications for health, education, and society. It manifests through impaired self-control, tolerance, and withdrawal, mirroring mechanisms seen in SUDs, and contributes to sleep disruption, visual and musculoskeletal strain, psychiatric comorbidities, and impaired social functioning. Although tools like Young's IAT and SAS aid assessment, the lack of standardised diagnostic criteria remains a challenge. Current interventions, including educational programs, parental strategies, CBT, mindfulness, and limited pharmacological options, offer partial benefit, but long-term efficacy is uncertain. With prevalence rising globally, especially among youth, there is an urgent need for robust, longitudinal, and culturally sensitive research to refine diagnostics, strengthen prevention, and develop effective, scalable interventions, including artificial intelligence-driven approaches, to foster healthier digital engagement in an increasingly connected world.

## References

[1] Dresp-Langley B, Hutt A (2022). Digital addiction and sleep. Int J Environ Res Public Health.

[2] Andreassen CS, Pallesen S (2014). Social network site addiction - an overview. Curr Pharm Des.

[3] (2025). Where we stand: screen time. HealthyChildren.org.

[4] Bull FC, Al-Ansari SS, Biddle S (2020). World Health Organization 2020 guidelines on physical activity and sedentary behaviour. Br J Sports Med.

[5] Twenge JM, Campbell WK (2018). Associations between screen time and lower psychological well-being among children and adolescents: evidence from a population-based study. Prev Med Rep.

[6] Hsu WY, Lin SS, Chang SM, Tseng YH, Chiu NY (2015). Examining the diagnostic criteria for internet addiction: expert validation. J Formos Med Assoc.

[7] Young KS (1998). Internet addiction: The emergence of a new clinical disorder. Cyberpsychol Behav.

[8] Griffiths M (2025). Technological addictions. Clin Psychol Forum.

[9] Darvesh N, Radhakrishnan A, Lachance CC (2020). Exploring the prevalence of gaming disorder and Internet gaming disorder: a rapid scoping review. Syst Rev.

[10] Vahia VN (2013). Diagnostic and statistical manual of mental disorders 5: a quick glance. Indian J Psychiatry.

[11] Panova T, Carbonell X (2018). Is smartphone addiction really an addiction?. J Behav Addict.

[12] Lee JY, Kim SY, Bae KY, Kim JM, Shin IS, Yoon JS, Kim SW (2018). Prevalence and risk factors for problematic Internet use among rural adolescents in Korea. Asia Pac Psychiatry.

[13] Joseph J, Varghese A, Vr V (2021). Prevalence of internet addiction among college students in the Indian setting: a systematic review and meta-analysis. Gen Psychiatr.

[14] Müller KW, Dreier M, Beutel ME, Duven E, Giralt S, Wölfling K (2016). A hidden type of internet addiction? Intense and addictive use of social networking sites in adolescents. Comput Hum Behav.

[15] Sun Y, Li Y, Bao Y (2020). Brief report: increased addictive internet and substance use behavior during the COVID‐19 pandemic in China. Am J Addict.

[16] Chi X, Liang K, Chen ST (2021). Mental health problems among Chinese adolescents during the COVID-19: the importance of nutrition and physical activity. Int J Clin Health Psychol.

[17] Lu P, Qiu J, Huang S (2025). Interventions for digital addiction: umbrella review of meta-analyses. J Med Internet Res.

[18] Shaw M, Black DW (2008). Internet addiction: definition, assessment, epidemiology and clinical management. CNS Drugs.

[19] Acharya S, Adhikari L, Khadka S, Paudel S, Kaphle M (2023). Internet addiction and its associated factors among undergraduate students in Kathmandu, Nepal. J Addict.

[20] Meng SQ, Cheng JL, Li YY (2022). Global prevalence of digital addiction in general population: a systematic review and meta-analysis. Clin Psychol Rev.

[21] Liu X, Gui Z, Chen ZM (2025). Global prevalence of internet addiction among university students: a systematic review and meta-analysis. Curr Opin Psychiatry.

[22] Cheng C, Li AY (2014). Internet addiction prevalence and quality of (real) life: a meta-analysis of 31 nations across seven world regions. Cyberpsychol Behav Soc Netw.

[23] Auxier B, Anderson M (2021). Social Media Use in 2021. https://www.pewresearch.org/wp-content/uploads/sites/20/2021/04/PI_2021.04.07_Social-Media-Use_FINAL.pdf.

[24] Király O, Potenza MN, Stein DJ (2020). Preventing problematic internet use during the COVID-19 pandemic: consensus guidance. Compr Psychiatry.

[25] Su W, Han X, Jin C, Yan Y, Potenza MN (2019). Are males more likely to be addicted to the internet than females? A meta-analysis involving 34 global jurisdictions. Comput Hum Behav.

[26] Mei S, Yau YH, Chai J, Guo J, Potenza MN (2016). Problematic Internet use, well-being, self-esteem and self-control: data from a high-school survey in China. Addict Behav.

[27] Tsitsika A, Janikian M, Schoenmakers TM (2014). Internet addictive behavior in adolescence: a cross-sectional study in seven European countries. Cyberpsychol Behav Soc Netw.

[28] Li D, Zhang W, Li X, Zhen S, Wang Y (2010). Stressful life events and problematic Internet use by adolescent females and males: a mediated moderation model. Comput Hum Behav.

[29] Sun P, Johnson CA, Palmer P (2012). Concurrent and predictive relationships between compulsive internet use and substance use: findings from vocational high school students in China and the USA. Int J Environ Res Public Health.

[30] Aylaz R, Güneş G, Günaydın Y, Kocaer M, Pehlivan E (2016). Problematic internet usage among high school students and the relevant factors. Turk J Public Health.

[31] Weinstein A, Lejoyeux M (2010). Internet addiction or excessive internet use. Am J Drug Alcohol Abuse.

[32] Ding K, Shen Y, Liu Q, Li H (2023). The effects of digital addiction on brain function and structure of children and adolescents: a scoping review. Healthcare (Basel).

[33] Brand M, Young KS, Laier C (2014). Prefrontal control and internet addiction: a theoretical model and review of neuropsychological and neuroimaging findings. Front Hum Neurosci.

[34] Li J, Yang H (2024). Unveiling the grip of mobile phone addiction: an in-depth review. Front Psychiatry.

[35] Weinstein A, Lejoyeux M (2020). Neurobiological mechanisms underlying internet gaming disorder. Dialogues Clin Neurosci.

[36] Li S, Wu Q, Tang C, Chen Z, Liu L (2020). Exercise-based interventions for internet addiction: neurobiological and neuropsychological evidence. Front Psychol.

[37] Sugaya N, Shirasaka T, Takahashi K, Kanda H (2019). Bio-psychosocial factors of children and adolescents with internet gaming disorder: a systematic review. Biopsychosoc Med.

[38] Casile A, Marraudino M, Bonaldo B, Micioni Di Bonaventura MV, Nasini S, Cifani C, Gotti S (2025). Novel rat model of gaming disorder: assessment of social reward and sex differences in behavior and c-Fos brain activity. Psychopharmacology (Berl).

[39] De D, El Jamal M, Aydemir E, Khera A (2025). Social media algorithms and teen addiction: neurophysiological impact and ethical considerations. Cureus.

[40] Seo HS, Jeong EK, Choi S, Kwon Y, Park HJ, Kim I (2020). Changes of neurotransmitters in youth with internet and smartphone addiction: a comparison with healthy controls and changes after cognitive behavioral therapy. AJNR Am J Neuroradiol.

[41] Weinstein A, Livny A, Weizman A (2017). New developments in brain research of internet and gaming disorder. Neurosci Biobehav Rev.

[42] Dores AR, Peixoto M, Fernandes C, Marques A, Barbosa F (2025). The effects of social feedback through the “like” feature on brain activity: a systematic review. Healthcare (Basel).

[43] Westbrook A, Ghosh A, van den Bosch R, Määttä JI, Hofmans L, Cools R (2021). Striatal dopamine synthesis capacity reflects smartphone social activity. iScience.

[44] He Q, Turel O, Bechara A (2017). Brain anatomy alterations associated with social networking site (SNS) addiction. Sci Rep.

[45] Kuss DJ, Pontes HM, Griffiths MD (2018). Neurobiological correlates in internet gaming disorder: a systematic literature review. Front Psychiatry.

[46] Horvath J, Mundinger C, Schmitgen MM (2020). Structural and functional correlates of smartphone addiction. Addict Behav.

[47] Pan N, Yang Y, Du X (2018). Brain structures associated with internet addiction tendency in adolescent online game players. Front Psychiatry.

[48] Yoo JH, Chun JW, Choi MR, Cho H, Kim JY, Choi J, Kim DJ (2021). Caudate nucleus volume mediates the link between glutamatergic neurotransmission and problematic smartphone use in youth. J Behav Addict.

[49] Sun JT, Hu B, Chen TQ (2023). Internet addiction-induced brain structure and function alterations: a systematic review and meta-analysis of voxel-based morphometry and resting-state functional connectivity studies. Brain Imaging Behav.

[50] Amirthalingam J, Khera A (2024). Understanding social media addiction: a deep dive. Cureus.

[51] Marsch LA (2020). Digital health and addiction. Curr Opin Syst Biol.

[52] Beyens I, Nathanson AI (2019). Electronic media use and sleep among preschoolers: evidence for time-shifted and less consolidated sleep. Health Commun.

[53] Kopilaš V, Korać D, Brajković L, Kopilaš M (2025). Visual functioning and mental health in the digital age. J Clin Med.

[54] Jakhar F, Rodrigues GR, Mendonca TM, Nayak RR, Kamath G, Kamath SJ, Kamath A (2023). Dry eye symptoms and digital eyestrain - emerging epidemics among university students due to online curriculum amid the COVID-19 pandemic. A cross-sectional study. Indian J Ophthalmol.

[55] Kang JH, Park RY, Lee SJ, Kim JY, Yoon SR, Jung KI (2012). The effect of the forward head posture on postural balance in long time computer based worker. Ann Rehabil Med.

[56] Seyedahmadi M, Rostami J, Khalaghi K (2025). The relationship between mobile phone addiction and changes in spinal column angles of male high school students with endomorphic body type. Int J Prev Med.

[57] Wacks Y, Weinstein AM (2021). Excessive smartphone use is associated with health problems in adolescents and young adults. Front Psychiatry.

[58] Jiotsa B, Naccache B, Duval M, Rocher B, Grall-Bronnec M (2021). Social media use and body image disorders: association between frequency of comparing one’s own physical appearance to that of people being followed on social media and body dissatisfaction and drive for thinness. Int J Environ Res Public Health.

[59] Johnson JG, Cohen P, Kasen S, Brook JS (2007). Extensive television viewing and the development of attention and learning difficulties during adolescence. Arch Pediatr Adolesc Med.

[60] Yen JY, Ko CH, Yen CF, Wu HY, Yang MJ (2007). The comorbid psychiatric symptoms of Internet addiction: attention deficit and hyperactivity disorder (ADHD), depression, social phobia, and hostility. J Adolesc Health.

[61] Santini ZI, Thygesen LC, Andersen S (2024). Social media addiction predicts compromised mental health as well as perceived and objective social isolation in denmark: a longitudinal analysis of a nationwide survey linked to register data. Int J Ment Health Addict.

[62] Laberge-Nadeau C, Maag U, Bellavance F, Lapierre SD, Desjardins D, Messier S, Saïdi A (2003). Wireless telephones and the risk of road crashes. Accid Anal Prev.

[63] McCartt AT, Hellinga LA, Bratiman KA (2006). Cell phones and driving: review of research. Traffic Inj Prev.

[64] Aleankushiu YFA, Radhi MM (2025). Digital addiction and its association with nutrition-related health behaviors among female secondary school students. Natl J Community Med.

[65] Caner N, Evgin D (2021). Digital risks and adolescents: the relationships between digital game addiction, emotional eating, and aggression. Int J Ment Health Nurs.

[66] Ma H, He JQ, Zou JM, Zhong Y (2021). Mobile phone addiction and its association with burnout in Chinese novice nurses: a cross-sectional survey. Nurs Open.

[67] Schulz van Endert T (2021). Addictive use of digital devices in young children: associations with delay discounting, self-control and academic performance. PLoS One.

[68] Block JJ (2008). Issues for DSM-V: internet addiction. Am J Psychiatry.

[69] Griffiths M (2000). Does internet and computer ‘addiction’ exist? Some case study evidence. Cyberpsychol Behav.

[70] Ko CH, Yen JY, Chen CC, Chen SH, Yen CF (2005). Proposed diagnostic criteria of Internet addiction for adolescents. J Nerv Ment Dis.

[71] Tao R, Huang X, Wang J, Zhang H, Zhang Y, Li M (2010). Proposed diagnostic criteria for internet addiction. Addiction.

[72] Kwon M, Lee JY, Won WY (2013). Development and validation of a Smartphone Addiction Scale (SAS). PLoS One.

[73] Kwon M, Kim DJ, Cho H, Yang S (2013). The smartphone addiction scale: development and validation of a short version for adolescents. PLoS One.

[74] Hamamura T, Kobayashi N, Oka T, Kawashima I, Sakai Y, Tanaka SC, Honjo M (2023). Validity, reliability, and correlates of the Smartphone Addiction Scale-Short Version among Japanese adults. BMC Psychol.

[75] Sharma MK, Anand N, Srivastava K, Sagar R, Marimuthu P, Roopesh BN, Saraswat S (2020). Mobile phone use screening test: development, validation, and implications for screening excessive mobile use. Ind Psychiatry J.

[76] Seema R, Heidmets M, Konstabel K, Varik-Maasik E (2022). Development and validation of the Digital Addiction Scale for Teenagers (DAST). J Psychoeduc Assess.

[77] Al-Mamun F, Mamun MA, ALmerab MM, Islam J, Gozal D, Muhit M (2025). Psychometric validation of the Bangla Digital Addiction Scale for Teenagers and its associated factors among adolescents: MeLiSA study. BJPsych Open.

[78] Bağatarhan T, Siyez DM (2023). The digital addiction scale for children: psychometric properties of the Turkish version. Curr Psychol.

[79] Xu J, Ma Y, Tan M, Lou J, Lu J, Zhou X (2025). Adaptation and validation of the Chinese version of the Digital Addiction Scale for Children (DASC). BMC Public Health.

[80] Manara CV, Mingolo S, Grassi M, Sors F, Prpic V, Agostini T, Murgia M (2024). The Abstinence from Smartphone Scale (ABSS- 10): psychometric properties and practical utility. Comput Hum Behav Rep.

[81] Lin YH, Chang LR, Lee YH, Tseng HW, Kuo TB, Chen SH (2014). Development and validation of the Smartphone Addiction Inventory (SPAI). PLoS One.

[82] Simó-Sanz C, Ballestar-Tarín MªL, Martínez-Sabater A (2018). Smartphone Addiction Inventory (SPAI): translation, adaptation and validation of the tool in Spanish adult population. PLoS One.

[83] Pavia L, Cavani P, Di Blasi M, Giordano C (2016). Smartphone Addiction Inventory (SPAI): psychometric properties and confirmatory factor analysis. Comput Hum Behav.

[84] Lin YH, Pan YC, Lin SH, Chen SH (2017). Development of short-form and screening cutoff point of the Smartphone Addiction Inventory (SPAI-SF). Int J Methods Psychiatr Res.

[85] Merlo LJ, Stone AM, Bibbey A (2013). Measuring problematic mobile phone use: development and preliminary psychometric properties of the PUMP scale. J Addict.

[86] Gansner M, Nisenson M, Carson N, Torous J (2020). A pilot study using ecological momentary assessment via smartphone application to identify adolescent problematic internet use. Psychiatry Res.

[87] Lissak G (2018). Adverse physiological and psychological effects of screen time on children and adolescents: literature review and case study. Environ Res.

[88] Theopilus Y, Al Mahmud A, Davis H, Octavia JR (2024). Preventive interventions for internet addiction in young children: systematic review. JMIR Ment Health.

[89] Walther B, Hanewinkel R, Morgenstern M (2014). Effects of a brief school-based media literacy intervention on digital media use in adolescents: cluster randomized controlled trial. Cyberpsychol Behav Soc Netw.

[90] Ozyurt G, Çağla Dinsever Eliküçük, Çalişkan Z, Evgin D (2017). Effects of triple p on digital technological device use in preschool children. J Child Fam Stud.

[91] Quaglia JT, Braun SE, Freeman SP, McDaniel MA, Brown KW (2016). Meta-analytic evidence for effects of mindfulness training on dimensions of self-reported dispositional mindfulness. Psychol Assess.

[92] Tomlinson ER, Yousaf O, Vittersø AD, Jones L (2018). Dispositional mindfulness and psychological health: a systematic review. Mindfulness (N Y).

[93] Cheng SS, Zhang CQ, Wu JQ (2020). Mindfulness and smartphone addiction before going to sleep among college students: the mediating roles of self-control and rumination. Clocks Sleep.

[94] Ayub S, Jain L, Parnia S (2023). Efficacy of cognitive behavioral therapy for internet addiction among adolescents and young adults: a systematic review of randomized controlled trials (RCTs). J Clin Med.

[95] Young KS (2007). Cognitive behavior therapy with internet addicts: treatment outcomes and implications. Cyberpsychol Behav.

[96] Horita H, Seki Y, Yamaguchi T, Shiko Y, Kawasaki Y, Shimizu E (2024). Videoconference-delivered cognitive behavioral therapy for parents of adolescents with internet addiction: pilot randomized controlled trial. JMIR Pediatr Parent.

[97] Ksiksou J, Maskour L, Alaoui S (2023). Effects of cognitive-behavioral group therapy on reducing levels of internet addiction, depression, anxiety, and stress among nursing students in morocco. Iran J Psychiatry Behav Sci.

[98] Pluhar E, Jhe G, Tsappis M, Bickham D, Rich M (2020). Adapting dialectical behavior therapy for treating problematic interactive media use. J Psychiatr Pract.

[99] Han DH, Renshaw PF (2012). Bupropion in the treatment of problematic online game play in patients with major depressive disorder. J Psychopharmacol.

[100] Song J, Park JH, Han DH (2016). Comparative study of the effects of bupropion and escitalopram on Internet gaming disorder. Psychiatry Clin Neurosci.

[101] Solly JE, Grant JE, Chamberlain SR (2022). Pharmacological interventions for problematic usage of the Internet (PUI): a narrative review of current progress and future directions. Curr Opin Behav Sci.

[102] (2025). Digital detox: what to know. https://www.webmd.com/balance/what-is-digital-detox.

[103] Marciano L, Jindal S, Viswanath K (2024). Digital detox and well-being. Pediatrics.

[104] Ramadhan RN, Rampengan DD, Yumnanisha DA (2024). Impacts of digital social media detox for mental health: a systematic review and meta-analysis. Narra J.

[105] Alanzi TM, Arif W, Aqeeli R (2024). Examining the impact of digital detox interventions on anxiety and depression levels among young adults. Cureus.

[106] Montag C, Duke É, Markowetz A (2016). Toward psychoinformatics: computer science meets psychology. Comput Math Methods Med.

[107] Brand M, Wegmann E, Stark R, Müller A, Wölfling K, Robbins TW, Potenza MN (2019). The Interaction of Person-Affect-Cognition-Execution (I-PACE) model for addictive behaviors: update, generalization to addictive behaviors beyond internet-use disorders, and specification of the process character of addictive behaviors. Neurosci Biobehav Rev.

